# Epigenome-wide association study of alcohol consumption in *N* = 8161 individuals and relevance to alcohol use disorder pathophysiology: identification of the cystine/glutamate transporter SLC7A11 as a top target

**DOI:** 10.1038/s41380-021-01378-6

**Published:** 2021-12-02

**Authors:** Falk W. Lohoff, Toni-Kim Clarke, Zachary A. Kaminsky, Rosie M. Walker, Mairead L. Bermingham, Jeesun Jung, Stewart W. Morris, Daniel Rosoff, Archie Campbell, Miruna Barbu, Katrin Charlet, Mark Adams, Jisoo Lee, David M. Howard, Emma M. O’Connell, Heather Whalley, David J. Porteous, Andrew M. McIntosh, Kathryn L. Evans

**Affiliations:** 1 Section on Clinical Genomics and Experimental Therapeutics, National Institute on Alcohol Abuse and Alcoholism, National Institutes of Health, Bethesda, MD USA; 2 Division of Psychiatry, University of Edinburgh, Royal Edinburgh Hospital, Edinburgh, UK; 3 Royal’s Institute of Mental Health Research, University of Ottawa, Ottawa, ON Canada; 4 Centre for Genomic and Experimental Medicine, Institute of Genetics and Cancer, University of Edinburgh, Edinburgh, UK; 5 Social, Genetic and Developmental Psychiatry Centre, Institute of Psychiatry, Psychology & Neuroscience, King’s College London, London, UK

**Keywords:** Addiction, Genetics

## Abstract

Alcohol misuse is common in many societies worldwide and is associated with extensive morbidity and mortality, often leading to alcohol use disorders (AUD) and alcohol-related end-organ damage. The underlying mechanisms contributing to the development of AUD are largely unknown; however, growing evidence suggests that alcohol consumption is strongly associated with alterations in DNA methylation. Identification of alcohol-associated methylomic variation might provide novel insights into pathophysiology and novel treatment targets for AUD. Here we performed the largest single-cohort epigenome-wide association study (EWAS) of alcohol consumption to date (*N* = 8161) and cross-validated findings in AUD populations with relevant endophenotypes, as well as alcohol-related animal models. Results showed 2504 CpGs significantly associated with alcohol consumption (Bonferroni *p* value < 6.8 × 10^−8^) with the five leading probes located in *SLC7A11* (*p* = 7.75 × 10^−108^)*, JDP2* (*p* = 1.44 × 10^−56^)*, GAS5* (*p* = 2.71 × 10^−47^)*, TRA2B* (*p* = 3.54 × 10^−42^), and *SLC43A1* (*p* = 1.18 × 10^−40^). Genes annotated to associated CpG sites are implicated in liver and brain function, the cellular response to alcohol and alcohol-associated diseases, including hypertension and Alzheimer’s disease. Two-sample Mendelian randomization confirmed the causal relationship of consumption on AUD risk (inverse variance weighted (IVW) *p* = 5.37 × 10^−09^). A methylation-based predictor of alcohol consumption was able to discriminate AUD cases in two independent cohorts (*p* = 6.32 × 10^−38^ and *p* = 5.41 × 10^−14^). The top EWAS probe cg06690548, located in the cystine/glutamate transporter SLC7A11, was replicated in an independent cohort of AUD and control participants (*N* = 615) and showed strong hypomethylation in AUD (*p* < 10^−17^). Decreased CpG methylation at this probe was consistently associated with clinical measures including increased heavy drinking days (*p* < 10^−4^), increased liver function enzymes (GGT (*p* = 1.03 × 10^−21^), ALT (*p* = 1.29 × 10^−6^), and AST (*p* = 1.97 × 10^−8^)) in individuals with AUD. Postmortem brain analyses documented increased *SLC7A11* expression in the frontal cortex of individuals with AUD and animal models showed marked increased expression in liver, suggesting a mechanism by which alcohol leads to hypomethylation-induced overexpression of SLC7A11. Taken together, our EWAS discovery sample and subsequent validation of the top probe in AUD suggest a strong role of abnormal glutamate signaling mediated by methylomic variation in SLC7A11. Our data are intriguing given the prominent role of glutamate signaling in brain and liver and might provide an important target for therapeutic intervention.

## Introduction

Alcohol use disorder (AUD) is a highly prevalent chronic relapsing disorder characterized by impaired ability to control or stop alcohol use [1]. Excessive alcohol use is a major risk factor for various cancers and organ damage, including alcohol-associated liver disease (ALD), and has been associated with cognitive impairment and progressive white matter degeneration in the brain. AUD has a complex pathophysiology and the exact biological mechanisms by which alcohol influences AUD and related diseases are unclear but likely include epigenetic mediation of environmental and genetic risk factors.

It is hypothesized that alcohol may influence disease outcomes through epigenetic modifications: alcohol is known to affect the acetylation and methylation of histones and the methylation of DNA [2]. DNA methylation involves the addition of a methyl group, donated by the metabolite, S-adenosylmethionine (SAM), to the C of CpG dinucleotides. Chronic alcohol consumption (AC) leads to a reduction in SAM, which can lead to hypomethylation across the epigenome [3]. Alcohol also impacts the folate cycle that is necessary for the generation of methionine for the synthesis of methyl groups [4] and the highly reactive alcohol metabolite acetaldehyde can induce inhibition of DNA methyltransferases, the family of enzymes that catalyze CpG methylation, to reduce methylation [5, 6]. Alcohol metabolism also acutely depletes molecules needed for re-methylation by increasing reactive oxygen species formation, which results in decreased production of methionine and SAM [7].

Epigenome-wide association studies (EWAS) have identified CpGs that are associated with AC [8–12] and AUD [13–16]. However, prior studies were somewhat limited by small sample sizes and lower capture arrays [16]. In addition, only a few studies exist linking AC and AUD EWAS data with detailed biological validation [8, 13, 15, 17].

To address these gaps in the literature, we conducted the largest EWAS analyses of AC in a single cohort of individuals (*n* = 8161) and followed up top findings in AUD-relevant phenotypes using a translational cross-tissue/cross-phenotypic approach to identify novel potential targets relevant to AUD.

## Materials and methods

### Cohorts

Generation Scotland: The Scottish Family Health Study (GS): GS is a family-based cohort comprising 24,069 individuals recruited from the general population of Scotland through General Practitioners/Primary Care Physicians [18, 19]. As part of a preclinical questionnaire, participants were asked about their drinking status (current, former, never) and current drinkers were asked to report the number of units of alcohol consumed in the previous week. Information regarding the typical number of units in different drink types was available to help participants calculate weekly intake [20]. Participants also reported their smoking status (never, former, current) and level of smoking (cigarettes per day). DNA was extracted from peripheral blood samples that were taken at the point of recruitment (2006–2011). Ethical approval for the GS study was obtained from the Tayside Committee on Medical Research Ethics (on behalf of the National Health Service). Demographic information and additional information can be found in Supplementary Tables S1–S11 and Supplementary Figs. 1–3.

Alcohol Use Disorder cohort 1: 539 participants (336 AUD, 203 controls) were recruited from the National Institute on Alcohol Abuse and Alcoholism (NIAAA) at the National Institutes of Health (NIH), USA. An alcohol-dependence (AD) diagnosis was made using the Structured Clinical Interview for Diagnostic and Statistical Manual of Mental Disorders (DSM)-IV-TR (SCID-IV) [21]. AD diagnosed using the DSM-IV corresponds to a moderate to severe AUD DSM-5 diagnosis [22]. Controls were recruited via the NIAAA Clinical Program. Subjects completed several self-report questionnaires and clinical assessments. Peripheral blood was obtained for DNA methylation analyses. Participants provided written informed consent in accordance with the Declaration of Helsinki and the study was approved by the NIAAA Institutional Review Board. Additional cohort information can be found elsewhere [15].

Alcohol Use Disorder cohort 2: 86 participants (43 AUD and 43 controls) were recruited by the NIAAA for a separate study on fear conditioning and extinction [23]. AUD was diagnosed according to the SCID-IV [21] with alcohol specified as the drug of choice and AC reported in the past 30 days. Exclusion criteria included self-reported neurological symptoms, chronic medication use (psychotropic or fluoxetine), DSM-IV diagnosis of bipolar disorder, psychotic disorder, or additional substance dependence other than nicotine or caffeine. Controls were screened for a history of seizures relating to alcohol withdrawal or alcohol withdrawal scores ≥8 on the Clinical Institute Withdrawal Assessment Alcohol Revised. Venous blood was collected for DNA methylation analyses. Participants provided written informed consent in accordance with the Declaration of Helsinki and the study was approved by the NIAAA Institutional Review Board.

### DNA methylation

Generation Scotland wave 1 (set 1): DNA methylation was assessed using the MethylationEPIC BeadChip in whole-blood-derived DNA from 5200 individuals. Quality control was performed using ShinyMethyl v.1.18.0 [24] to visually inspect the log median intensity of methylated vs. unmethylated signal per array and remove outliers. WateRmelon v 1.26.0 [25] was used to remove any samples in which ≥1% of CpG dinucleotides had a detection *p* value in excess of 0.05, where probes with a beadcount of less than 3 in more than 5 samples was observed, and any probes in which ≥0.5% of samples had a detection *p* value in excess of 0.05. Samples were also removed if predicted sex did not match recorded sex, leaving 5087 samples available for analysis. Due to the presence of closely related individuals within the wave 1 sample, *M* values were pre-corrected for relatedness, array processing batch, and estimated cell counts.

Generation Scotland wave 2 (set 2): DNA methylation was assessed using the MethylationEPIC BeadChip in whole-blood-derived DNA from 4574 unrelated individuals. Individual in wave 2 were unrelated to individuals in wave 1. Similar quality control criteria were applied to wave 2 [26], which left 4450 samples for analysis. The data were normalized using the dasen function in wateRmelon v 1.26.0 [25] and normalized *M* values produced using the getM function.

Alcohol Use Disorder cohorts: DNA methylation data from whole blood samples were assessed using the MethylationEPIC BeadChip microarray. Methylation data were processed using the wateRmelon package [25] and any probes that were predicted to cross-react and/or that failed QC were removed. The dasen method in the wateRmelon package was used to quantile-normalize methylated and unmethylated intensities in the red and green channels separately, followed by beta-value calculation.

### EWAS analyses

Generation Scotland: in wave 1, each CpG was fitted as the dependent variable and, using linear regression models run in the limma R package, tested for their association with log-transformed (+1) units of alcohol per week. Age, sex, smoking status, pack years, and the first 20 principal components from the corrected *M* values were fitted as fixed effect covariates. The same approach was used for the GS wave 2 data, but batch and estimated cell counts (CD4, CD8, granulocytes, B and NK cells) were fitted as fixed effect covariates as these were not adjusted for in the normalization process. Probes were excluded if there were any individuals with missing data at that CpG.

In wave 1 there were 4301 current drinkers available with methylation data after QC and 3860 current drinkers available in wave 2. Meta-analysis of these two datasets was performed using sample-size weighted *p* value based analysis in METAL [27] (*n* = 8161). There were 731,208 CpGs available after QC across both waves available for meta-analysis and a Bonferroni correction (0.05/731,208) was used to define epigenome-wide significance (*p* ≤ 6.8 × 10^−8^). Sensitivity analyses were carried out by running an EWAS of AC in never smokers. There were 2218 never smokers available for analysis in wave 1 and 2020 in wave 2 (current and former smokers excluded). The same statistical model was used for the EWAS, although smoking status and pack years were not fitted.

### Identification of differentially methylated regions

Differentially methylated regions (DMR), defined as regions containing 2-30 CpG sites separated by ≤500 bp, were identified using the dmrff.meta function implemented in the dmrff R package [28]. CpGs with unadjusted EWAS meta-analysis *p* values ≤ 0.05 and methylation changes in a consistent direction were used for DMR analysis and DMRs with a Bonferroni-adjusted *p* value ≤ 0.05 declared statistically significant.

### Gene annotation

The GENE2FUNC function in FUMA (https://fuma.ctglab.nl) [29, 30] was used to provide biological annotation. FUMA interrogates sets of differentially expressed genes (DEG): sets of genes which are more (or less) expressed in a specific tissue compared to other tissue types based on GTEx RNA-seq data. Hypergeometric tests were used to evaluate whether the differentially methylated genes were overrepresented in DEG sets from the 30 general and 54 specific tissue types in the GTEx v8 database.

### Two-sample Mendelian randomization analyses

We performed bidirectional two-sample MR analyses of AD and AC using publicly available GWAS summary-level data from the Psychiatric Genomics Consortium (PGC) and the GWAS and Sequencing Consortium of Alcohol and Nicotine Use (GSCAN), respectively, both meta-analyzing cohorts of predominantly European ancestry. Both GWAS studies have existing ethical permissions from their respective institutional review boards and include participant informed consent and included rigorous quality control.

For purposes of the PGC GWAS, AD was defined as meeting criteria of the DSM-IV (or DSM-IIIR in one instance) diagnosis of AD (cases *N* = 8485; controls *N* = 20,657) [31]. AC was measured in terms of drinks per week (DPW) across 28 cohorts (*N* = 537,249): given the disparate measurement methods across cohorts (binned, normalized, etc.), the data were log-transformed; the effect estimate is, therefore, measured in log-transformed DPW [32]. For the analysis of AC on AD, we included all SNPs associated with AC at *p* < 5 × 10^−8^ and pruned with the stringent pairwise linkage disequilibrium (LD) *r*^2^ < 0.001 (to ensure validity and statistical independence), leaving 37 SNPs. For the analysis of AD on AC, we included all SNPs associated with AD at *p* < 5 × 10^−6^ and again pruned with the stringent pairwise LD *r*^2^ < 0.001 (to ensure validity and statistical independence), leaving 18 SNPs. We extracted summary statistics for the bidirectional instruments for AC and AD, from the AD and AC GWASs, correspondingly, then harmonized effect alleles, and removed SNPs that were palindromic with intermediate allele frequency (Supplementary Table S12a, b).

We report results from IVW MR and complementary MR–Egger, weighted median, and weighted mode MR methods to assess potential causality between AC and AD: consistency of results across methods strengthens an inference of causality [33] (Supplementary Table S13 and Supplementary Fig. S4A, C). To evaluate potential heterogeneity, we used the MR–Egger intercept test [34], the Cochran heterogeneity test [35], and the Mendelian randomization pleiotropy residual sum and outlier (MR-PRESSO) global test to identify and remove outlier SNPs to correct potential directional horizontal pleiotropy and resolve detected heterogeneity. We used the Steiger directionality test to test the causal direction between the hypothesized exposure and outcomes [36]. In addition, we used leave-one-out analyses to assess whether high leverage points have high influence (Supplementary Fig. S4B, D). Our analysis was carried out using TwoSampleMR, version 5.5 [33] and MR-PRESSO, version 1.0 [37] in the R environment, version 4.0.2.

Since sample overlap between the PGC and GSCAN GWAS cohorts was minimal (0.8%), and instrument strength considered strong (mean F statistic, AD, 24.5, AC, 74.0, exceeding conventional threshold 10), considerable weak instrument bias is not expected (Supplementary Table S12a, b) [38]. We found evidence of an association between genetic liability for AC and increased risk for AD (odds ratio = 5.55 per standard deviation increase in log-transformed DPW, 95% CI, 2.65–11.62, *p* = 5.41 × 10^−6^); however, we did not find evidence of an association in the reverse direction between genetic liability of increased risk for AD and AC (beta = 0.008, 95% CI, −0.003 to 0.18, *p* = 0.17) (Supplementary Table S13). The associations were similar in magnitude and direction across the four complementary MR methods (Supplementary Fig. S4A, C), with no residual heterogeneity, bias due to pleiotropy, or apparent points of high leverage have high influence (Supplementary Fig. S4B, D).

### AUD methylation risk score analyses using LASSO regression

DNA methylation predictors were trained on the Generation Scotland quality controlled, pre-normalized beta values using the R package *biglasso*. Probes were restricted to only those available on the Illumina 450 K array in order to maximize the use of the methylation predictor in other cohorts that may only have 450 K data. AC in current drinkers was first residualized for age, sex and ten genetic principal components and then penalized regression applied using the “cv.biglasso” function and ten-fold cross-validation. Non-zero coefficients from this model, with the lambda value corresponding to the mean square error, were taken to create methylation risk scores (MRS) for AC in the two independent AUD cohorts.

For each individual in AUD Cohorts 1 and 2, an AUD risk score was generated by taking the sum of the product of 519 DNA methylation beta values and 519 supplied model coefficient values. Using the pROC package in R, the prediction accuracy was assessed by calculating the area under the receiver operator characteristic curve (AUC) against the AUD diagnostic status as defined above.

### Analysis of cg06690548 in the National Institute on Alcohol Abuse and Alcoholism (NIAAA)

The sample consists of 615 participants, 372 with AUD and 243 healthy controls (HC). All participants provided a blood sample that was used for genome-wide DNA methylation analysis and clinical biomarker collection, including the liver function tests for GGT, ALT, and AST. The lipid measurements for low-density lipoprotein cholesterol (LDL-C), high-density lipoprotein cholesterol (HDL-C), total cholesterol, and triglycerides (TG) were measured using standard procedures in mg/dL. Participants completed the DSM-IV-TR SCID-IV to determine an AD diagnosis. Participants also completed self-report questionnaires including the Timeline Followback, a measure of alcohol intake over the previous 90 days, and the Fagerström test for nicotine dependence. All participants completed screening assessments where information on their demographics and recent drinking history was collected. A subset of 86 (43 AUD and 43 HC) participants from the sample was recruited for a study on fear conditioning and extinction in AUD [23]. All study participants were recruited to the NIAAA at the NIH, USA. All participants provided informed written consent in accordance with the Declaration of Helsinki and were compensated for their time. The study was approved by the Institutional Review Board of the NIAAA.

DNA methylation levels from whole blood samples were assessed using an Infinium MethylationEPIC BeadChip microarray (Illumina Inc., San Diego, California) according to the manufacturer’s protocol. The wateRmelon package in R was used to process the raw data. After cross-reactive probes and probes that failed quality assessment were removed, a scale-based correction was applied for Illumina type I relative to type II probes. We used a quantile normalization approach to make methylated and unmethylated intensity values identical and then quantified the β-value using the ratio of intensities between methylated and unmethylated alleles. The *β* value of cg06690548 located in promoter of *SLC7A11* was extracted for further analysis [39].

To examine differences in methylation of cg06690548 between AUD and HC, a logistic regression model was utilized with adjustments for age, sex, and race. In addition, a linear regression model was used with liver function enzymes, lipid measurements, and total drinks after natural log transformation to satisfy normality as a dependent variable and cg06690548 as an independent variable. Age, sex, race, and AUD diagnosis as covariates were adjusted for. Number of drinking days and heavy drinking days were analyzed using a poisson regression model with the same covariate adjustments. All analyses were performed using Statistical Analysis System (version 9.4; SAS Institute Inc., Cary, NC). Significant differences were determined statistically at *p* < 0.05.

### Targeted mRNA analysis of liver tissues from rodents fed an alcohol liquid diet and controls

Two-month-old Sprague–Dawley male rats were obtained from Charles River (Kingston, NY). The animals were housed in a room with a 12 h/12 h light/dark cycle and had continuous access to food and water before the beginning of the experiment. The rats were given water and a nutritionally balanced liquid diet as the sole source of calories in their home cages (ad libitum). The detailed composition of alcohol liquid diet and experimental paradigm were stated in Lee et al. [40]. Briefly explaining, the rats received 5% of ethanol during a 5-day acclimation period, and liquid diet that contained 12% of ethanol was thereafter given for 6 weeks for the alcohol groups. For the control group, mixture of sweetener and sucrose was used for an isocaloric match to the alcohol liquid diet. All animal experiments were approved by the NIAAA Animal Care and Use Committee. All procedures were performed in accordance with the guidelines of the NIH Guide for the Care and Use of Laboratory Animals.

Rat tissues were homogenized in TRI Reagent^®^ (Zymo Research Corp) with ceramic beads using Precellys 24 Homogenizer at 4–8 °C. RNAs were isolated using Direct-zol™ RNA MiniPrep kit according to manufacturer’s instruction (Zymo Research Corp). cDNAs were prepared using High-Capacity cDNA Reverse Transcription Kit (Thermo Fisher Scientific) from those isolated RNAs. Real-time quantitative polymerase chain reaction (PCR) was run in ViiA™ 7 Real-Time PCR System using TaqMan Gene Expression Assays (SLC7A11: Rn01495125_m1, Thermo Fisher). The expression levels of target genes were normalized to the housekeeping gene (Glyceraldehyde-3-phosphate dehydrogenase: Rn01775763_g1, Thermo Fisher) and calculated based on the comparative cycle threshold Ct method (2−ΔΔCt). The data were expressed as mean ± SEM, and sample number represents the number of animals. Mann–Whitney *U* test was used for non-normally distributed data. The data analysis was performed using GraphPad prism 8.0 (GraphPad Software Inc., San Diego, CA, USA). Significant differences were determined statistically at *p* < 0.05.

### Targeted human postmortem brain mRNA analysis

Postmortem tissues were obtained from the New South Wales Tissue Resource Centre at the University of Sydney, Australia. Brain tissues from 11 males with AUD and 13 male controls were analyzed for prefrontal cortex (PFC). All AUD subjects had alcohol detected in blood and were also daily smokers at the time of death. Total RNA was extracted from male postmortem frozen brain tissue, using the RNeasy Lipid Tissue mini Kit (Qiagen). One microgram total RNA was reverse-transcribed using SuperScript^®^ III First-Strand Synthesis SuperMix for qRT-PCR (Invitrogen). Real-time quantitative PCR was run in ViiA™ 7 Real-Time PCR System using TaqMan Gene Expression Assays (SLC7A11: Hs00204938_ml, Thermo Fisher). The data were expressed as mean ± SEM, and sample number represents the number of individuals. Mann–Whitney *U* test was used for non-normally distributed data. The data analysis was performed using GraphPad prism 8.0 (GraphPad Software Inc., San Diego, CA, USA). Significant differences were determined statistically at *p* < 0.05.

## Results

### EWAS of alcohol consumption

**Fig. 1:**
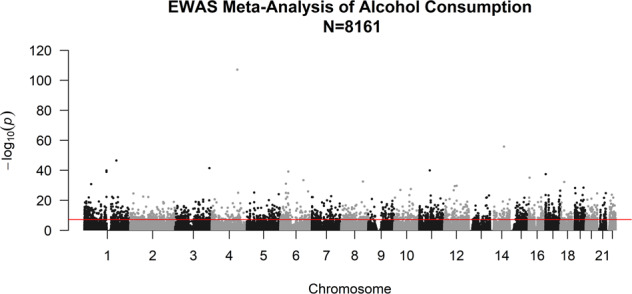
Manhattan plot showing EWAS of alcohol consumption meta-analysis. Line defines the threshold for epigenome-wide significance (*p* ≤ 6.8 × 10^−8^).

We used two discovery cohorts: Generation Scotland wave 1 (*N* = 5087 after QC) and Generation Scotland wave 2 (*N* = 4450 after QC). A final sample for a meta-analysis comprised *N* = 8161 individuals. Details can be found in the eMethods. There were 4301 current drinkers in the wave 1 EWAS and 3860 current drinkers in wave 2, the mean AC in each wave was 10.7 (SD = 11.2) and 10.9 (SD = 11.0) units per week, respectively. Additional descriptive and demographic variables for each wave are presented in Supplementary Table S1. Meta-analysis across the two waves (*N* = 8161) identified 2504 CpGs that were associated with AC (*p* ≤ 6.8 × 10^−8^). The top 20 CpGs are shown in Table 1 and all epigenome-wide significant CpGs from the meta-analysis are listed in Supplementary Table S2 and depicted as a Manhattan plot in Fig. 1. A detailed table of the top 20 CpGs and their association in wave 1 and wave 2 is shown in Supplementary Table S3.

**Table 1:** Top 20 CpGs associated with alcohol consumption in the meta-analysis (*N* = 8161) along with gene annotations, full gene names, chromosome, and base-pair position of the CpG (Ensembl (v92)).

CpG	Chrom	bp	Gene	Gene name/description	Direction	*Z* score	*p*
cg06690548	4	139162808	*SLC7A11*	Cystine/glutamate transporter	–	−22.06	7.75E–108
cg06088069	14	75895604	*JDP2*	Jun dimerization protein 2	–	−15.849	1.44E–056
cg06644515	1	173834831	*GAS5*	Growth arrest-specific 5	–	−14.445	2.71E–047
cg12825509	3	185648568	*TRA2B*	Transformer-2 protein homolog beta	–	−13.61	3.54E–042
cg11376147	11	57261198	*SLC43A1*	Large neutral amino acids transporter small subunit 3	–	−13.35	1.18E–040
cg14476101	1	120255992	*PHGDH*	Phosphoglycerate dehydrogenase	–	−13.34	1.33E–040
cg18120259	6	43894639	*LOC100132354*		–	−13.226	6.19E–040
cg26457483	1	120256112	*PHGDH*	Phosphoglycerate dehydrogenase	–	−13.185	1.07E–039
cg12116137	17	1576449	*PRPF8*	Pre-mRNA-processing-splicing factor 8	++	12.92	3.48E–038
cg03497652	16	4751569	*ANKS3*	Ankyrin Repeat and Sterile Alpha Motif Domain Containing 3	++	12.49	8.12E–036
cg25124205	6	125519976	*TPD52L1*	Tumor protein D53	–	−12.187	3.65E–034
cg15837522	8	117892654			–	−12.021	2.74E–033
cg16113793	18	21451607	*LAMA3*	Laminin subunit alpha 3	++	11.96	5.78E–033
cg01538969	6	30624636	*DHX16*	Putative pre-mRNA-splicing factor ATP-dependent RNA helicase	++	11.731	8.80E–032
cg10254445	1	37197260			–	−11.684	1.54E–031
cg21912872	12	68055270	*DYRK2*	Dual specificity tyrosine-phosphorylation-regulated kinase 2	–	−11.462	2.06E–030
cg08228578	12	57624193	*SHMT2*	Serine hydroxymethyltransferase	–	−11.428	3.04E–030
cg02711608	19	47287964	*SLC1A5*	Neutral amino acid transporter B(0)	–	−11.211	3.61E–029
cg12973487	19	1623075	*TCF3*	Transcription factor 3	++	11.177	5.30E–029
cg01307228	10	90152007	*RNLS*	Renalase	–	−11.03	2.76E–28

**Fig. 2:**
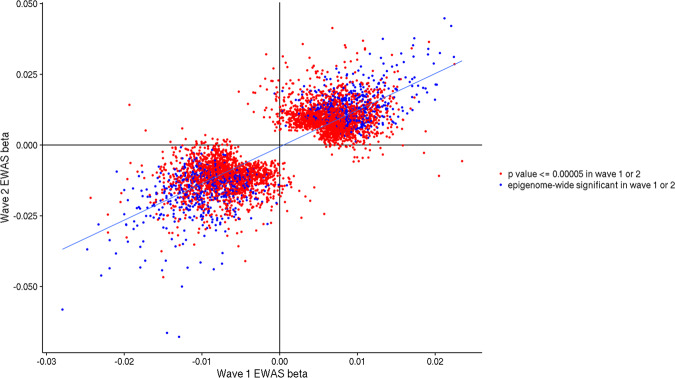
Correlation of effect sizes (beta) for CpGs in the wave 1 and wave 2 EWAS. CpG sites associated with alcohol consumption with a *p* value ≤ 5 × 10^−5^ in either wave 1 or wave 2 are shown in red. CpG sites associated with alcohol consumption with a *p* value ≤ 6.8 × 10^−8^ in either wave are shown in blue. For ease of plotting, cg06690548 is excluded due to its larger effect size in the EWAS (cg06690548: wave 1 beta = −0.069; wave 2 beta = −0.106).

Pearson’s correlation between the EWAS effect sizes from wave 1 and wave 2 across all CpGs was *r* = 0.138 (95% CI = 0.135–0.140). When restricting the CpGs to those with a *p* value of ≤5.0 × 10^−5^ in either the wave 1 or wave 2 EWAS, the correlation of effect sizes was *r* = 0.828 (95% CI = 0.819–0.837). For CpGs that reached epigenome-wide significance in either wave 1 or wave 2, the correlation between effect sizes increased to 0.90 (95% CI = 0.887–0.911) (Fig. 2).

Due to the substantial comorbidity between alcohol use and smoking, and the strong impact of smoking on DNA methylation, sensitivity analyses were performed in both wave 1 and wave 2 in the subset of individuals who had never smoked. Across all CpGs in wave 1 the correlation between effect sizes in the total alcohol EWAS and the non-smokers EWAS was *r* = 0.713 (95% CI = 0.712–0.714). Restricting to the CpGs associated with alcohol use with a significance cut off of *p* ≤ 5.0 × 10^−5^ the correlation increased to *r* = 0.961 (95% CI = 0.958–0.964). Similarly, in wave 2, the correlation across all CpGs was *r* = 0.722 (95% CI = 0.712–0.714) and *r* = 0.961 (95% CI = 0.959–0.964) when restricting to CpGs significant at *p* ≤ 5.0 × 10^−5^ (Supplementary Fig. S1).

The 2504 CpGs associated with AC in the meta-analysis mapped to 1510 unique genes, 1409 of which were found to have a recognized Ensembl (v92) ID. The GENE2FUNC function in FUMA [29, 30] was used to provide biological annotation of these genes. For the general tissue types, the differentially methylated genes were overrepresented amongst those genes normally expressed at low levels in the pancreas, heart and liver (Supplementary Fig. S2). For the specific tissue types, the alcohol-associated differentially methylated genes were significantly enriched for genes amongst those expressed at lower levels in sub-regions of the above tissues plus sub-regions of the brain (Supplementary Fig. S3). Gene Ontology (GO) biological processes (Supplementary Table S4), Kyoto Encyclopedia of Genes and Genomes (KEGG) pathways (Supplementary Table S5), and curated gene sets (Supplementary Table S6) were also tested for enrichment using FUMA. The top two significant GO processes were locomotion and regulation of intracellular signal transduction. The top KEGG pathways enriched for differentially methylated genes were the regulation of the actin cytoskeleton and pathways in cancer. The curated gene sets that showed most significant enrichment for our differentially methylated genes included a set of genes down regulated in erythroid progenitors from fetal liver in a mouse model knock-out of KLF1 [41] and a set of genes upregulated in the brain of patients with Alzheimer’s disease [42].

DMR, spanning 2 or more CpGs, were also identified. Five hundred and thirty-six DMRs ranging from 2–9 CpGs in length were found to be associated with AC using the meta-analysis *p* values (Supplementary Table S7). The most significantly associated region spanned 2 CpGs in the first intron of the *PHGDH* gene, a region also identified in the single CpG analysis. The largest DMR was located on chromosome 16 and spans 9 CpGs (1243 bp) at the MYC-associated zinc finger protein (*MAZ*) locus (*p* = 1.9 × 10^−4^), this region has a single CpG associated at the level of individual probes (cg03527802, *p* = 4.2 × 10^−9^).

The EWAS catalog (http://www.ewascatalog.org/) was used to cross-reference the 2504 CpGs significantly associated with AC with the wider literature (Supplementary Table S8). Overlap was found with 56 individual studies with traits including those related to AC [43], development and ageing (in liver, brain, and blood tissue) [42, 44–48], smoking [49–51], cancer [52, 53], the immune system [54, 55], and metabolic phenotypes [56–58].

### Two-sample Mendelian randomization analyses of alcohol consumption and AUD

We next investigated whether identified AC EWAS signals might be relevant to AUD pathophysiology, and to establish the validity of an AC derived MRS as a biomarker for AUD risk (see below). We therefore used publicly available GWAS summary-level data to conduct bidirectional two-sample Mendelian randomization analyses to formally determine whether there was evidence for a causal relationship between AC and AUD. Conceptually, Mendelian randomization has similarities with randomized controlled trials, with randomization of genetic variants occurring at meiosis [59, 60]. This approach is an additional strategy for strengthening causal inference when randomized controlled trials are impractical or unethical [61–63].

We found evidence of an association between genetic liability for AC and increased risk for AUD (odds ratio = 5.55 per standard deviation increase in log-transformed DPW, 95% CI, 2.65–11.62, *p* = 5.41 × 10^−6^); however, we did not find evidence of an association in the reverse direction, i.e., between genetic liability for increased risk for AUD and AC (beta = 0.008, 95% CI, −0.003 to 0.18, *p* = 0.17) (Supplementary Tables S12a, b and S13). The associations were similar in magnitude and direction across the four complementary Mendelian randomization methods (Supplementary Fig. S4A, C), with no residual heterogeneity, bias due to pleiotropy, nor apparent points of high leverage (Supplementary Fig. S4B, D).

### DNA methylation risk scores (MRS) for alcohol consumption and AUD diagnosis

**Fig. 3:**
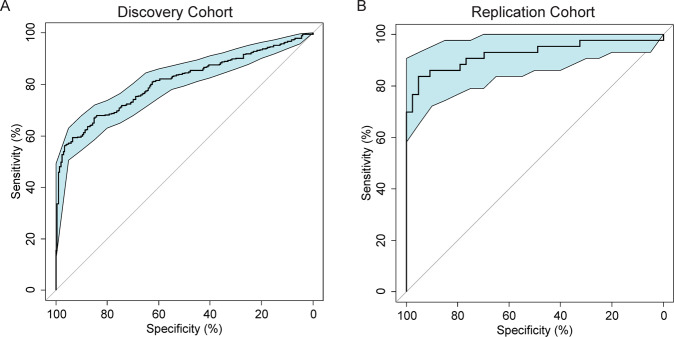
AUD methylation risk score analyses in AUD cohorts. Receiver operating characteristic curves for the methylation risk score prediction of AUD in Cohort 1 (**A** left) and in Cohort 2 (**B** right).

Having determined that there is evidence for a relationship between genetic liability for AC and AUD diagnosis, we developed a DNA MRS for AC and tested prediction of an AUD diagnosis in an independent cohort. LASSO regression of the Generation Scotland methylation data selected 519 CpGs to create a MRS for AC. The full list of CpG and their corresponding weights are listed in Supplementary Table S9. The MRS was then used to predict AUD in two independent cohorts of mixed ethnicity collected in the USA (AUD cohorts 1 and 2, Supplementary Tables S10 and S11). In cohort 1, the prediction scores were significantly associated with AUD status in a linear model adjusted for age, sex, and race (*b* = 0.59 + 0.042, *F* = 97.71, *R*^2^ = 0.42, df = 4/533, *p* = 6.32 × 10^−38^), and predicted AUD status with an AUC of 0.81 (95% CI: 0.77–0.84, Fig. 3). In cohort 2, a similarly significant association with AUD status was observed in a linear model, controlling for age, sex and race (*b* = 0.88 + 0.096, *F* = 20.07, *R*^2^ = 0.64, df = 7/78, *p* = 5.41 × 10^−14^). Prediction of AUD status using the prediction score achieved an AUC of 0.92 (95% CI: 0.86–0.99, Fig. 3). Although cohort 2 was a smaller sample, there were stricter inclusion and exclusion criteria for assignment of case-control status, which may explain the increased discriminatory power of the predictor in this cohort. Additional analyses separated by ethnicity and sex revealed similar AUC (Supplementary Table S14).

### SLC7A11 cg06690548 methylation and AUD

We next tested whether blood-based methylation for the top CpG from our discovery EWAS of AC was also associated with clinically diagnosed AUD in an independent cohort (see Supplementary Table S15 for details). Probe cg06690548 was significantly hypomethylated in AUD patients (*n* = 372) compared to HC (*n* = 243) after adjustment for age, gender, and ethnicity (*p* = 4.1E–17; mean cg06690548 methylation 0.75 (0.004) for the AUD group and 0.81 (0.002) for the controls by a logistic regression model after adjustment for age, gender, and ethnicity). Investigation of the AUD and control samples also showed that AC, including indications of severity, such as the number of drinking days and heavy drinking days, was associated with hypomethylation of the CpG site cg06690548 (Table 2).

**Table 2:** Association of *SLC7A11* cg06690548 methylation with clinical phenotypes, liver enzymes, and lipids.

	Total sample (*N* = 615)				AUD-only subset (*N* = 372)			
Variables	Beta	SE	STAT	*p* value	Beta	SE	STAT	*p* value
Total drinks	−1.31	0.80	−1.63	0.104	−1.17	0.55	−2.15	0.0325
Number of drinking days	−0.84	0.07	118.53^a^	<0.0001	−0.86	0.08	117.29^a^	<0.0001
Heavy drinking days	−1.15	0.08	195.09^a^	<0.0001	−1.09	0.08	172.10^a^	<0.0001
GGT	−5.46	0.55	−9.95	1.03E–21	−5.63	0.69	−8.18	1.67E–15
ALT	−2.08	0.42	−4.89	1.29E–06	−2.06	0.51	−4.07	5.32E–05
AST	−2.50	0.44	−5.69	1.97E–08	−2.47	0.57	−4.37	1.46E–05
HDL-cholesterol	0.16	0.24	0.7	0.4851	0.21	0.28	0.74	0.4619
LDL-cholesterol	−25.22	24.19	−1.04	0.2975	−37.09	27.27	−1.36	0.1747
Total cholesterol	−0.37	0.14	−2.65	0.0082	−0.45	0.16	−2.82	0.0051
Triglycerides	−1.47	0.35	−4.2	3.07E–05	−1.68	0.41	−4.13	4.13E–05

^a^Wald *χ*^2^ statistic was estimated by a poisson regression model.

### cg06690548 methylation and liver and lipid profiles

Given the strong association of alcohol related phenotypes with CpG sites located close to SLC7A11, we next examined whether reduced cg06690548 methylation was associated with clinically relevant liver- and lipid-related phenotypes. In the total sample of 615 participants, decreased level of cg06690548 methylation was associated with significantly increased levels of the liver enzymes GGT (beta = −5.46; *p* = 1.03E–21), ALT (beta = −2.08; *p* = 1.29E–06), and AST (beta = −2.50; *p* = 1.97E–08) as seen in Table 2. In addition, hypomethylation at cg06690548 was associated with increased total cholesterol (beta = −0.37; *p* = 0.0082) and TG levels (beta = −1.47; *p* = 3.07E–05) but was not associated with LDL-C or HDL-C levels.

### Hepatic SLC7A11 expression in a liquid diet rat model

**Fig. 4:**
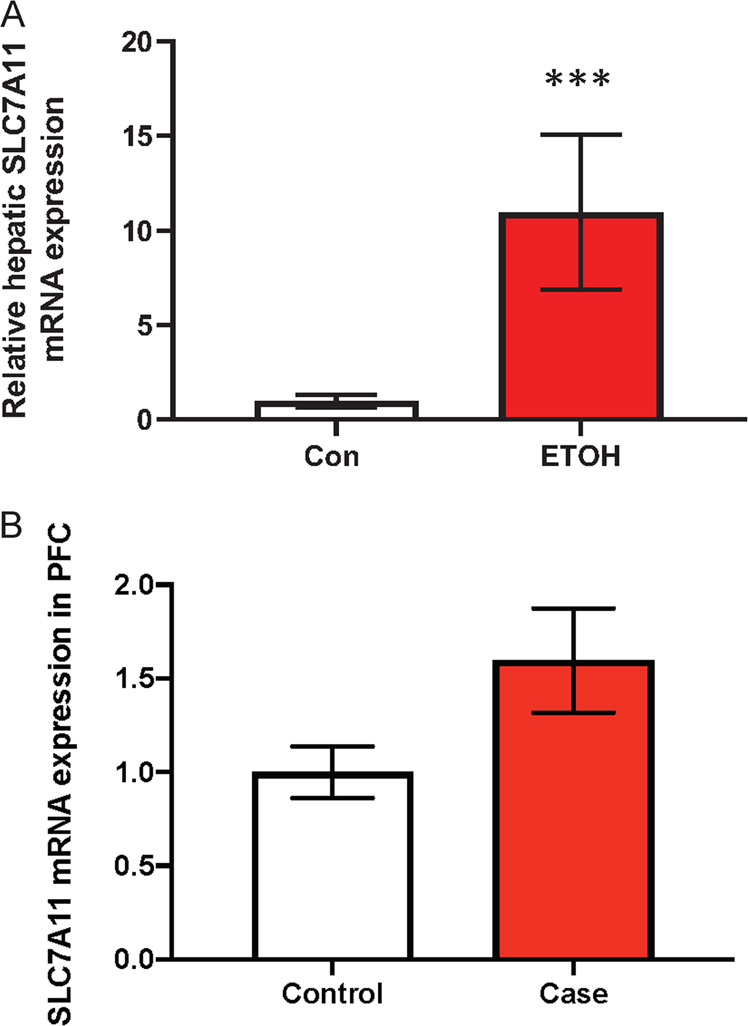
SLC7A11 experssion in rat liver and human postmortem brain. **A** Effect of alcohol intake on SLC7A11 expression in the liver of rats. Relative hepatic mRNA expression in the chronic alcohol-fed rat model (*n* = 7 for control and 8 for ethanol groups). **B** Effect of alcohol consumption on SLC7A11 expression in human postmortem brain. Relative hepatic mRNA expression in PFC of individuals with AUD (*n* = 13 for control and 11 for case).

Given the association of *SLC7A11* methylation with AUD and hepatic and lipidemic phenotypes, we next investigated whether chronic alcohol intake influenced the level of SLC7A11 expression in the liver of alcohol-fed rats. In this rat model, it was previously shown that chronic alcohol exposure induced hepatocellular injury, hepatic inflammation, and TG accumulation [40]. In line with the association analyses, chronic alcohol exposure significantly increased SLC7A11 mRNA levels in the rat liver (*p* = 0.0006) (Fig. 4A). In order to further investigate the effects of promoter DNA methylation at SLC7A11 on expression, we downloaded publicly available datasets of rat liver generated by MeDIP-Seq (GSE53518) and Affymetrix Rat Genome 230 2.0 Arrays (GSE51797), respectively. The primary data set was generated to assess pharmacologically induced carcinoma, which may influence hepatic cell-type ratios, so we confined our analysis to *N* = 4 water-treated control rat livers. A significant negative association was observed between promoter DNA methylation and gene expression of SLC7A11 (*R* = −0.97, *p* = 0.029), suggesting that the hypomethylation observed in our study has the potential to be linked with the observed expression upregulation, although future larger studies are needed to confirm this effect.

### SLC7A11 expression in human postmortem brain

We also analyzed expression of SLC7A11 in individuals with AUD and controls using human postmortem brain tissues. SLC7A11 mRNA level expression in PFC of individuals with AUD significantly was increased compared to the controls (*p* = 0.0352) (Fig. 4B).

## Discussion

In this paper, we carried out the largest EWAS of AC so far, conducted MR analyses to establish a link between AC and AUD risk, followed up with the generation of a biological methylation prediction score based on AC data for AUD risk, and validated the top probe in *SLC7A11* in various animal and human endophenotypic datasets.

### EWAS and target validation

We report multiple associations between DNA methylation and AC in the Generation Scotland cohort. There was a strong correlation between waves 1 and 2 of the Generation Scotland cohort and sensitivity analyses indicated that the results were not substantially confounded by smoking.

The most significant association with AC was with cg06690548, a CpG site located in the promoter region of *SLC7A11*, which has previously been reported to be associated with increased AC [10, 11, 14, 43, 64]. In addition, in an EWAS of liver enzyme levels, this probe was the top hit for association with ALT and GGT levels and was also associated with a reduced risk of hepatic steatosis [65].

The *SLC7A11* gene encodes the light chain (xCT) of the cystine/glutamate antiporter (system x_c_^–^) that transports the anionic form of cystine into cells in exchange for glutamate. System x_c_^–^ is expressed in several cells including macrophages, hepatocytes, endothelial cells, neurons, astrocytes, and microglia [66]. The exported glutamate makes up the extracellular glutamate concentration in the brain [66–69]. System x_c_^–^ plays an important role in preventing oxidative stress and damage in cells, as the imported cystine is reduced to cysteine, the rate-limiting precursor for synthesis of glutathione (GSH). This mechanism is crucial for both brain and liver. In brain, a recent study showed that cg06690548 hypermethylation in Parkinson’s disease was associated with downregulation of the *SLC7A11* gene and reduced GSH levels and increased oxidative stress triggered degeneration of dopaminergic neurons in the substantia nigra [70]. We found that decreased methylation at cg06690548 was associated with AC in both the population-based cohorts and AUD cohorts. Subsequent biological validation of the effects of alcohol on SLC7A11 methylation and expression using a chronic rat model and human postmortem brain confirmed that decreased methylation at cg06690548 leads to increased mRNA expression, further supporting a functional role of this SLC7A11 CpG site. Our data are in line with a recent study by Choi et al. who showed that alcohol increases xCT/SLC7A11 expression in liver in mice and patients with ALD leading to increased extracellular glutamate levels in the liver [71]. Genetic or pharmacologic inhibition of xCT attenuated alcoholic steatosis in mice, highlighting the potential for therapeutic intervention. Similarly, alcohol exposure consistently upregulated xCT/SLC7A11 transporter expression in rat brain [72], consistent with our EWAS data of hypomethylated cg06690548.

Our endophenotypic analysis of alcohol-associated phenotypes showed that various liver biomarkers were robustly associated with SLC7A11 methylation status. This finding is intriguing, as a recent EWAS on NAFLD also identified SLC7A11 methylation status as risk factor for hepatic steatosis [73]. In addition, postmortem brain analyses showed that SLC7A11 was upregulated in key brain regions previously implicated in AUD, highlighting potential direct effects on brain structure and physiology. Thus, our study makes an important contribution to the growing evidence that SLC7A11 is an important molecule that spans the liver–brain-axis in alcohol-related disease and may represent a novel therapeutic target for these conditions. While there are currently no FDA-approved medications that selectively target SLC7A11/xCT, there is growing interest in SLC7A11 inhibitors in the cancer field, as SLC7A11 overexpression promotes tumor growth partially by suppressing ferroptosis, which might also be relevant to alcohol-related liver cancers [74, 75]. Interestingly, N-acetylcysteine, a cytosine prodrug that targets SLC7A11/xCT decreases synaptic glutamate transmission and oxidative stress and has been investigated in several preclinical and clinical studies as a potential therapeutic target for psychiatric and substance use disorders [76–78].

There were several other candidate genes that were also strongly associated with AC in our EWAS with potential implication on alcohol-associated disease. Three CpGs located in the gene encoding the phosphoglycerate dehydrogenase (*PHGDH*) showed statistically significant differential methylation in our study. This finding is important given that the cg14476101 site has been previously been associated with high blood pressure [79], which is often a consequence of excessive chronic alcohol use [80] and normalizes for the majority of individuals after AC cessation [81, 82]. A large meta-analysis found that reducing AC lowers blood pressure in a dose-dependent manner [83]. Interestingly, differential methylation at multiple genes (*SLC7A11*, *PHGDH*, *TXNIP*, *LOC100132354*, *CPT1A*, *SLC1A5*) is associated with both AC (this study) and with hypertension [79].

The c-Jun-dimerization protein 2 (*JPD2*) locus contains four CpGs associated with AC. *JDP2* is a component of the AP-1 transcription factor that has been shown to be involved in the development of liver cancer in mice [84]. Four CpGs at the *GAS5* locus were also associated with AC. The top CpG (cg06644515) was also associated with AUD and alcohol use in a previous study of three independent cohorts [13]. GAS5 methylation is associated with morning cortisol levels, suggesting a role for HPA-axis regulation in the development of AUD [13].

The differentially methylated genes were overrepresented amongst genes usually expressed at low levels in some of the key tissues impacted by alcohol usage (liver, pancreas, and brain). Enrichment was also seen amongst genes whose upregulation is associated with Alzheimer’s disease, and the longest DMR we discovered, which is in the MAZ gene, is amongst this gene set and is known to localize to pathologic structures in Alzheimer’s disease brain [85]. These data support the link observed between AC and Alzheimer’s disease [86, 87].

### Mendelian randomization of alcohol consumption and AUD

While there has been substantial literature that chronic heavy AC can increase the risk for AUD, observational epidemiological data are prone to confounding and reversed causation making causal inference and directionality difficult. In order to investigate the relationship between AC and AUD risk, we performed a two-sample bidirectional Mendelian randomization analysis. Our data show evidence for a potential causal association between the genetic liability for increase AC and the risk for AUD. Although GWAS analyses by Walters et al. did not find overwhelming genetic overlap between AC phenotypes and AUD, there are several GWAS limitations that might contribute to this “lack of finding” of overlap, including phenotypic considerations and lack of power and study design. Mendelian Randomization is a complementary genetic approach, and in this case, we used AC as exposure and AUD as outcome, with non-overlapping samples. It may well be the case that future larger AUD GWAS studies will show a greater overlap of genetic factors influencing AC with genetic risk variants for AUD. Our Mendelian randomization finding suggests that the underlying biological mechanism for heavy AC might also be relevant in the pathophysiology of AUD. Based on these results we further analyzed if AC MRSs can predict AUD risk.

### Methylation risk scores and AUD risk

Using MRSs trained on the Generation Scotland cohort, we were able to discriminate between AUD cases and controls in two independent USA-based cohorts of mixed ethnicity. Given that the AUC for AUD in the present study was as high as 0.92, this DNA methylation signature may have clinical utility as a biomarker for heavy drinking. This is comparable, and indeed better, than many existing clinical biomarkers for alcohol use, such as elevated liver enzymes or carbohydrate-deficient transferrin [88]. While measurement of phosphatidylethanol (PEth), an objective measure of AC, can be considered currently the gold-standard of assessing AC compared to self-report measures [89], PEth also has some limitation including a half-life of ~4–7 days [90] and a limited window of detection of about 21 days [91]. In this regard, a MRS for AC might offer a longer-term biomarker signature profile, although future studies are needed to test how stable this measure is over time [8] and how clinically useful it might be.

Previous studies that have attempted to create DNA methylation-based biomarkers of alcohol use have not been as successful in discriminating heavy drinkers from controls. McCartney et al., using a subset of the Generation Scotland cohort used in this study, were able to predict heavy drinking in an independent population-based cohort with an AUC of 0.73 [92]. A study by Liu et al. developed a predictor [43] that when tested in independent cohorts predicted heavy drinking with maximum AUCs of 0.60 and 0.77 and problem drinking with a maximum AUC of 0.80 [93]. As studies of methylation-based biomarkers for alcohol use were found to be less predictive in adolescents compared to adults [93], it is likely that the methylation scores represent exposure to long-term alcohol use. This measure may be of use as a proxy for self-reported alcohol use when these measures are unavailable, or to complement self-report when the measurement reliability is questioned. Further work is needed to determine the stability of alcohol-associated methylation after drinking cessation, as this will inform the clinical applicability of MRSs as biomarkers.

### Strengths and limitations

We note several strengths of our study, including the largest single sample cohort EWAS to date, providing adequate power to detect a large range of effects and enabling a high degree of precision. As a result, we identified a range of genes with robust statistical evidence for an association with AC. In addition, we carried out biological target validation studies, which is a crucial step in moving a large set of data findings to meaningful and potentially clinically important new target identification, as shown with SLC7A11. Finally, by using Mendelian randomization to validate the use of AC phenotypes as surrogate for AUD risk, we calculated a methylation prediction score that robustly identified AUD status, highlighting the usefulness of EWAS approaches for validating disease diagnostics and monitoring.

There are also several limitations that should be carefully taken into consideration when interpreting our results. We describe DNA methylation changes observed in peripheral blood in our EWAS, which may not be informative for the many other tissues impacted by AC. To address this limitation, we have carried out several replication and biological validation experiments for the top target SLC7A11, including brain and liver as well as animal cohorts. Interestingly, we observed convergence of evidence that *SLC7A11* might be relevant to alcohol related pathology in both liver and brain and were able to detect this target using a peripheral blood DNA EWAS approach. While studies of blood are essential for developing effective biomarkers for clinical use and for discovery of potential new biological targets, the exact mechanisms by which alcohol leads to widespread epigenetic modifications are not addressed in our study. Furthermore, while it is likely that alcohol exposure leads to DNA methylation changes, it is possible that some DNA methylation changes can also lead to changes in AC behaviors. Longitudinal studies are, therefore, warranted.

We also note that our discovery cohort was predominantly a population-based sample of EA ancestry, limiting inference to other populations and possible disease populations. Future studies need to include other ethnic groups and should also carry out sex-specific analyses. In addition, it is important to note that there are several other factors besides AC that can have effects on the epigenetic landscape, such as phenotypic heterogeneity, various environmental factors including life-experiences and lifestyle and underlying genetic architecture.

### Summary

In summary, we describe an EWAS of AC in 8161 individuals in a population-based cohort. We find 2504 CpGs where DNA methylation is significantly associated with levels of AC and identify several enriched gene pathways and gene sets. Biological validation and endophenotypic studies identified SLC7A11 as a top target relevant to liver and brain, and other genes related to often observed clinical comorbidities of chronic heavy AC, including hypertension and Alzheimer’s disease. This research serves as a proof-of-principle study showing that upstream phenotypes, such as AC, can have direct relevance to underlying disease. Future studies are needed to determine the clinical utility of methylation biomarkers as proxies for alcohol exposure and to establish the degree to which they can aide in the discovery of novel therapeutic targets for AUD and alcohol-related diseases.

## Supplementary information

Supplementary

Supplementary tables
