# Supplementary material for: Epigenome-wide association study of alcohol consumption in N = 8161 individuals and relevance to alcohol use disorder pathophysiology: identification of the cystine/glutamate transporter SLC7A11 as a top target
Source: Mol Psychiatry. 2021 Dec 2;27(3):1754–64. doi: 10.1038/s41380-021-01378-6 (PMC9095480; doi:10.1038/s41380-021-01378-6)
Supplement: Supplementary file 1 — Supplementary [file 41380_2021_1378_MOESM1_ESM.docx]

**Supplementary**

*Epigenome-wide association study of alcohol consumption in N=8161 individuals and relevance for alcohol use disorder pathophysiology: identification of the cystine/glutamate transporter SLC7A11 as top target*

**Supplementary EWAS on Alcohol Consumption** **Tables S1-S11** (in the excel file)

**Supplementary** **Mendelian randomization (MR) Tables S12-S13** (in the excel file)

**Supplementary MRS for alcohol consumption and AUD diagnosis Table S14** (in excel file)

**Supplementary AUD cohort replication sample Table S15**

**Table S15. Demographic description of the NIAAA sample.**

| **N=615** | **AUD (N=372)** | **HC (N=243)** | **P-value** |
| --- | --- | --- | --- |
| Age (y), Mean (SE) | 44.52 (0.57) | 35.41 (0.80) | <0.0001 |
| Male, N (%) | 224 (60.22) | 118 (48.56) | 0.005 |
| Race, N (%) |  |  | 0.14 |
| Whites | 191 (51.34) | 133 (54.73) |  |
| Blacks | 177 (47.58) | 103 (42.39) |  |
| Others | 4 (1.08) | 7 (2.88) |  |
| GGT, Mean (SE) | 141.50 (12.2) | 26.05 (1.77) | <0.0001 |
| ALT, Mean (SE) | 50.32 (2.28) | 23.54 (1.23) | <0.0001 |
| AST, Mean (SE) | 53.70 (3.33) | 21.39 (1.01) | <0.0001 |
| Total Drinks, Mean (SE) | 937.52 (36.44) | 51.33 (7.29) | <0.0001 |
| Number of Drinking Days, Mean (SE) | 72.95 (1.10) | 18.58 (1.38) | <0.0001 |
| Heavy Drinking Days, Mean (SE) | 64.08 (1.43) | 3.51 (0.77) | <0.0001 |
| Fagerstrom test for Nicotine Dependence Score, Mean (SE) | 2.3 (0.13) | 0.12 (0.03) | <0.0001 |
| Fagerstrom Test for Nicotine Dependence(%) | 206 (55.4) | 8 (3.3) | <0.0001 |
| Major Depression Recurrent, (%) | 73 (20.7) | 2(0.88) | <0.0001 |
| Major Depression Single, (%) | 32 (9.1) | 17(7.5) | 0.54 |
| Opioid Dependence, (%) | 30(8.5) | 0 (0) | <0.0001 |
| Cocaine Dependence, (%) | 91(25.8) | 0 (0) | <0.0001 |
| Cannabis Dependence, (%) | 70 (19.3) | 4 (1.75) | <0.0001 |

**Figure S1**: Sensitivity analyses comparing the EWAS of alcohol consumption in the total sample to an EWAS of alcohol consumption in never smokers. A: Wave 1 correlation B: Wave 2 correlation.


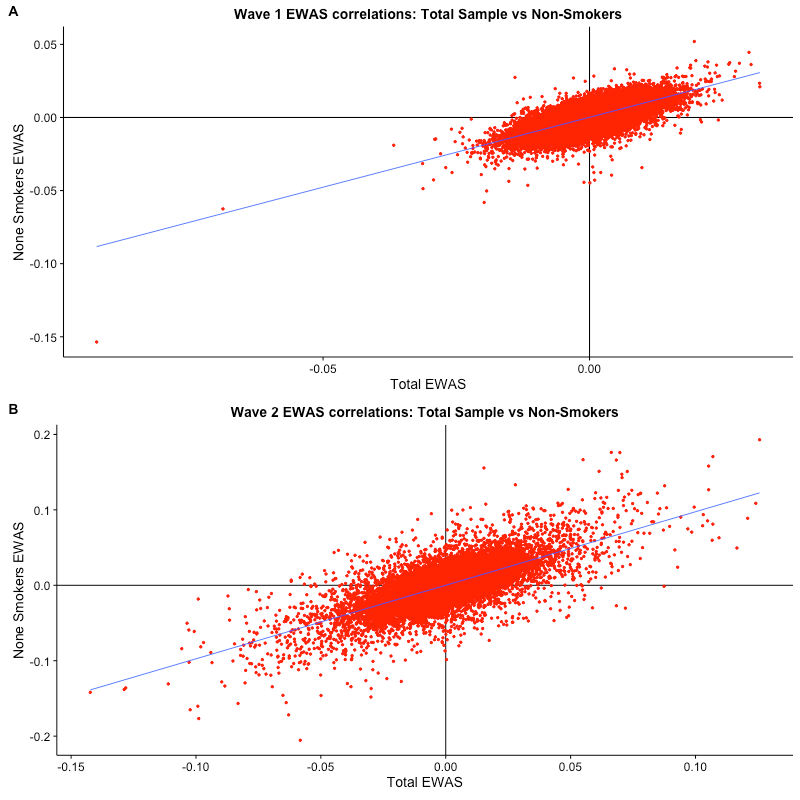


**Figure S2**: Enrichment of genes containing a CpG showing differential methylation related to alcohol consumption in the EWAS amongst differentially expressed genes (DEGs) across 30 different tissue types from GTEx v8 (https://fuma.ctglab.nl). Red indicates Bonferroni corrected P-value ≤ 0.05.


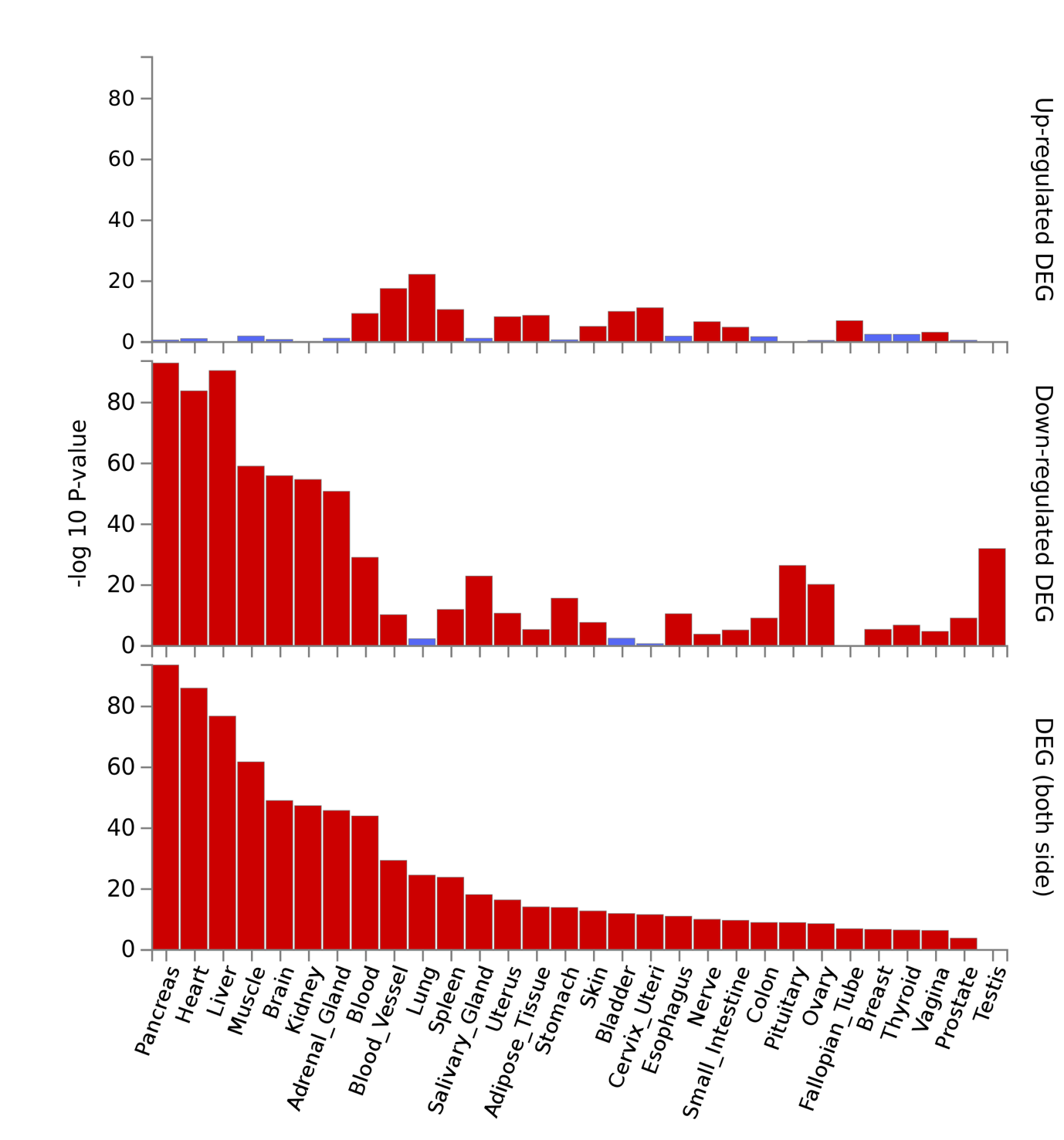


**Figure S3**: Enrichment of genes containing a CpG showing differential methylation related to alcohol consumption in the EWAS amongst differentially expressed genes (DEGs) across 54 specific tissue types from GTEx v8 (https://fuma.ctglab.nl). Red indicates Bonferroni corrected P-value ≤ 0.05.


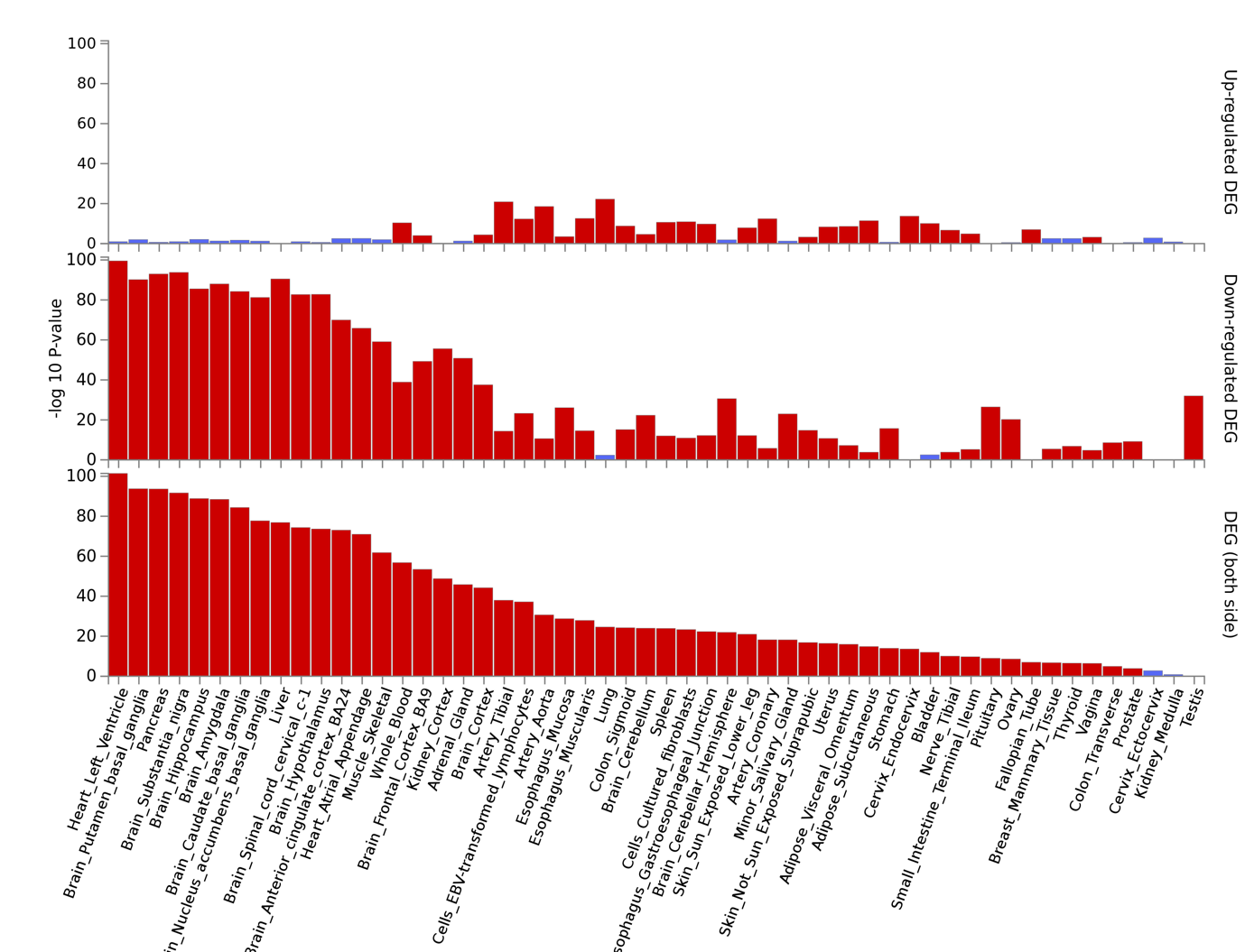


**Figure S4.** **Scatterplot and leave-one-out analysis of bidirectional associations of genetic liability of alcohol consumption (drinks per week) and risk of alcohol dependence.** (A, C) Scatterplot of independent instrument SNP exposure effects versus outcome effects from two independent samples augmented by the standard error of these effects on the vertical and horizontal sides (for presentation, alleles are coded so that all SNP exposure effects are positive) for (A) alcohol consumption on risk of alcohol dependence and (C) genetic risk of alcohol dependence on alcohol consumption. Solid lines are the regression slopes fitted by the primary MR IVW and four complementary MR methods: slopes fitted by MR IVW method were similar in direction and magnitude to slopes fitted by MR Egger and weighted median methods. (B, D) In leave-one-out analyses, MR IVW was performed leaving out each SNP in turn to determine whether any single SNP may be driving the association, with results illustrated in plots showing that no SNP was driving the association between (B) genetic risk of alcohol consumption on risk of alcohol dependence nor (D) risk of alcohol dependence on consumption. Heterogeneity tests did not indicate heterogeneity, and pleiotropy robust methods did not indicate bias in the MR IVW estimates (**Table S13 in the Supplement**).

**Abbreviations:** MR, Mendelian randomization; SNP, single nucleotide polymorphism, here selected as instruments.

(A)

(B)

(C)

(D)
